# Roles of Sedentary Behaviors and Unhealthy Foods in Increasing the Obesity Risk in Adult Men and Women: A Cross-Sectional National Study

**DOI:** 10.3390/nu10060704

**Published:** 2018-05-31

**Authors:** Esti Nurwanti, Mohy Uddin, Jung-Su Chang, Hamam Hadi, Shabbir Syed-Abdul, Emily Chia-Yu Su, Aldilas Achmad Nursetyo, Jakir Hossain Bhuiyan Masud, Chyi-Huey Bai

**Affiliations:** 1 International PhD Program in Medicine, College of Medicine, Taipei Medical University, Taipei 11031, Taiwan; estinurwanti3@gmail.com; 2 Department of Nutrition, Faculty of Health Science, Universitas Alma Ata, Yogyakarta 55183, Indonesia; hamamhadi99@gmail.com; 3 King Abdullah International Medical Research Center, King Saud bin Abdul Aziz University for Health Sciences, Executive Office, King Abdul Aziz Medical City, Ministry of National Guard Health Affairs, Riyadh 11426, Kingdom of Saudi Arabia; mrmohy@gmail.com; 4 School of Nutrition and Health Sciences, College of Nutrition, Taipei Medical University, Taipei 11031, Taiwan; susanchang@tmu.edu.tw; 5 Graduate Institute of Metabolism and Obesity Sciences, College of Nutrition, Taipei Medical University, Taipei 11031, Taiwan; 6 Graduate Institute of Biomedical Informatics, College of Medical Science and Technology, Taipei Medical University, Taipei 10675, Taiwan; drshabbir@tmu.edu.tw (S.-S.A.); emilysu@tmu.edu.tw (E.C.-Y.S.); mail.aldilas@gmail.com (A.A.N.); jakirmsd@gmail.com (J.H.B.M.); 7 Department of Public Health, College of Medicine, Taipei Medical University, Taipei 11031, Taiwan; 8 School of Public Health, College of Public Health, Taipei Medical University, Taipei 11031, Taiwan

**Keywords:** obesity, sedentary behaviors, refined carbohydrates, sweet foods and beverages, fatty fried foods

## Abstract

Sedentary behaviors and dietary intake are independently associated with obesity risk. In the literature, only a few studies have investigated gender differences for such associations. The present study aims to assess the association of sedentary behaviors and unhealthy foods intake with obesity in men and women in a comparative manner. The analysis presented in this study was based on the data from a population-based, cross-sectional, nationally representative survey (Indonesian Basic Health Research 2013/RISKESDAS 2013). In total, 222,650 men and 248,590 women aged 19–55 years were enrolled. A validated questionnaire, physical activity card, and food card were used for the assessments. The results showed that the prevalence of obesity (body mass index of ≥27.5 kg/m^2^) was higher in women (18.71%) than in men (8.67%). The mean body mass index in women tended to be higher than in men. After adjusting for age and education, the gender effect on obesity persisted in women and was more significant than in men. There was also a positive and significant effect on obesity of sedentary behaviors and unhealthy foods intake. Moreover, fatty and fried foods displayed a positive multiplicative interaction, increasing obesity risk in women more than in men and indicating a possible dietary risk in in women in relation to obesity. The study suggests that the implementation of educational programs on nutrition and physical activity is particularly important for promoting a healthy body weight among Indonesian women.

## 1. Introduction

Obesity has become a major problem in developing countries and third-world countries in Southeast Asia, including Indonesia. Not only it has increased the risk of non-communicable diseases, such as hypertension, diabetes, dyslipidemia, and renal dysfunction [1], but also it has an important effect on the morbidity and mortality of the Asian population [2]. The combined prevalence of overweight and obesity among Indonesian adults aged 19–55 years increased from 19.8% in 2007 to 23% in 2010, especially in women (24.6% and 28.7%) compared to men (14.8% and 17%) in both years, respectively [3].

Changes in the dietary patterns and decreased physical activity are considered the most important determining factors of overweight and obesity in Indonesia [3,4]. Individuals who spent more time in sedentary activities and were not sufficiently physically active were shown to have an increased likelihood of being overweight or obese [5,6]. People with sedentary behaviors had a 3.7-fold greater chance of becoming obese than those who had limited sedentary behaviors and performed sufficient physical activity [7]. Meanwhile, there was a weak relationship between sedentary behaviors and moderate to vigorous physical activity [8,9,10,11,12]. Physical activity that varies in intensity may be differentially associated with body fat mass and distribution [13]. Although, sedentary behavior or a single dietary component might not directly increase body weight in an individual [14,15], several studies reported that unhealthy foods, such as refined carbohydrates [16], sweet foods and beverages [15,17], and fatty fried foods [18,19], increase the risk of obesity.

Evidence showed a strong increase in overweight and obesity prevalence in the female population [3]. Gender differences were reported in the relationship between dietary patterns and obesity in the middle-aged and elderly population [20], but a low level of physical activity was associated with a risk of being overweight or obese only in men [21]. Understanding possible gender and age-related differences in sedentary behaviors and dietary patterns are crucial to prevent obesity.

Therefore, using nationally representative data from 2013 Indonesia Basic Health Research (Riskesdas/Riset Kesehatan Dasar), we first compared the prevalence of obesity, the sedentary behaviors, and unhealthy foods intake in Indonesian adult men and women of different ages and then explored the associations of sedentary behaviors and unhealthy foods intake with obesity in the two genders.

## 2. Materials and Methods

### 2.1. Data Sources

We used a population-based, cross-sectional, nationally representative survey (Indonesia Basic Health Research 2013/Riskesdas 2013/Riset Kesehatan Dasar 2013) conducted by the National Institute of Health Research Development (NIHRD), Ministry of Health, Indonesia. A two-stage stratified cluster sampling method was used to draw the study sample from 33 provinces of the 497 Indonesian districts. The sampling and survey methods have been described in detail (website) [22]. Complete interviews were conducted for 294,959 households from the targeted 300,000 households (98.3%).

The 1,027,763 participants were aged from 0 months to 75 years in the national survey, but our study only focused on 518,111 participants aged 19–55 years. Out of those, 46,871 (9.05%) adults who were underweight (BMI < 18.5) or morbidly obese (BMI > 40) were excluded and, finally, 471,240 (90.95%) adults were included in the study.

The protocol, study design, data, and survey were reviewed and approved (LB.02.01/5.2/KE.006/2013) by the Health Research Ethics Committee—University of Indonesia, Hasanuddin University, the University of Airlangga, and The National Institute of Health Research and Development (NIHRD), Ministry of Health Republic of Indonesia.

### 2.2. Measurements

Basic characteristics and anthropometric measurements (height and weight) were collected on the basis of a standardized protocol by well-trained interviewers. The standing height was measured by a multi-function brand Stadiometer with a capacity of 2 m and a precision of 0.1 cm. The body weight was measured by a Camry digital weight scale with a capacity of 150 kg. The weight scale was calibrated daily before using it. The body mass index (BMI) was calculated as weight (kg)/height squared (m^2^) and was determined on the basis of the World Health Organization (WHO) criteria for the Asian population: normal weight (18.5 to <23 kg/m^2^), overweight (23.0 to <27.5 kg/m^2^), and obese (≥27.5 kg/m^2^) [23]. Several self-reported covariates were collected through interviews: age, gender (men and women), education (non-college graduates and college graduates).

The respondents were invited to recall all activities beginning from waking up in the morning until sleeping at night and were assessed for their habits of daily sedentary activities with a physical activity card and a questionnaire during one week. The interviewer recorded the type and time of an activity carried out continuously for ≥10 min. An very long time (more than 6 h) was further investigated for certainty. The data were excluded if a respondent was sick or could not move. The activities were: (1) a job in a paid and unpaid work environment, domestic work, harvesting agricultural products, fishing or animal hunting, searching for work, etc.; (2) relaxing time, including sports and recreation; (3) travel (walking or riding a bike) to the workplace, marketplace, or for recreation. Once recorded, all activities were grouped according to the type of physical activity. Sedentary behavior was defined as (i) sitting at work or home (ii); sitting for transport (including waiting for transport); (iii) sitting or lying down to watch television or play electronic games; (iv) sitting or lying down to do other activities (such as talking, reading, etc.), not including sleeping (or napping). The duration of the sedentary activities was recorded in hours or minutes. Similar to another study in our domain [24], sedentary activities were coded as <3 h, 3–5 h, or ≥6 h.

The respondents were asked about the frequency of refined carbohydrates, fatty fried foods, sweet foods, and beverages intake in the last week using a questionnaire and food card. Food frequency was recorded as >one time (1x)/day, 1x/day, 3–6x/week, 1–2x/week, ≤3x/month, and never. It was categorized in a binary form: frequently (≥1x/day) and infrequently (<1x/day) [22]. In food questionnaires, refined carbohydrates included processed foods of flour with added sugar, such as flavored bread. Sweet foods and beverages included high-sugar foods and beverages with additional natural sugar, e.g., cakes, canned fruit, artificial juice drinks, soft drinks, syrups, and sweet tea. Soft drinks and sweetened beverages with zero calories and low-sugar and diet drinks were excluded from this foods group. Fatty and fried foods included high-fat foods, e.g., fatty meats, oxtail soup, fried foods, foods containing coconut milk and margarine, and high-cholesterol foods, such as innards (intestines, tripe), eggs, and shrimp.

### 2.3. Statistical Analysis

**Figure 1:**
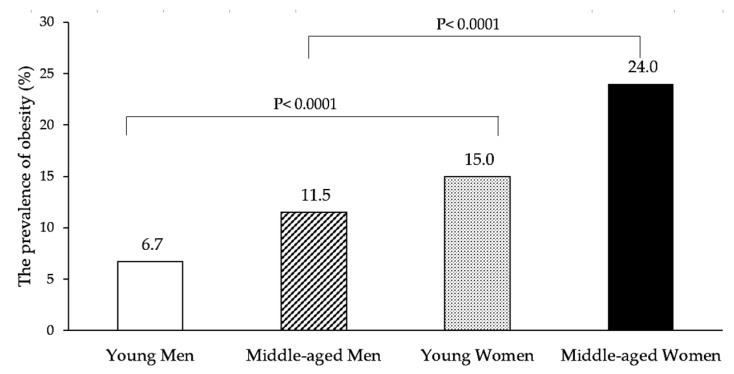
The prevalence of obesity (BMI ≥ 27.5 kg/m^2^) among Indonesian adults.

**Figure 2:**
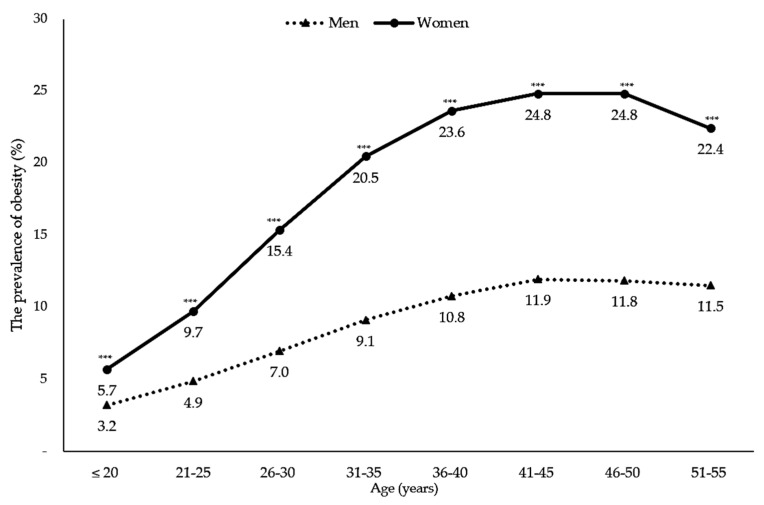
The prevalence of obesity by age. Footnote: *** *p* value < 0.0001.

Pearson’s Chi-squared test was used to test the obesity status, using age groups, gender, education, sedentary activities, and dietary intake as categorical variables (see Table 1, Figure 1 and Figure 2). Mean and standard deviation were used in continuous data, e.g., age, and examined by two-sample t-test or analysis of variance. The prevalence ratio with 95% confidence interval (CI) of sedentary behaviors and dietary intake for obesity risk was evaluated by Poisson regression. The SAS software (version. 9.4, Cary, NC, USA) was used for data analysis. The statistical threshold for significance was considered at *p* ≤ 0.05.

## 3. Results

The characteristics of all participants stratified by age and gender are reported in Table 1. The table shows that the mean body mass index was higher in women than in men in both the young aged group and the middle-aged group. In general, significantly more women were obese, with higher sedentary behavior and higher intake of refined carbohydrates and fatty and fried foods.

In terms of sedentary behaviors, increased sedentary habits were found both in young and in middle-aged women compared to men. Similar observations were made for the proportions of refined carbohydrates and fatty and fried foods habitually consumed, but not for sweet foods and beverages. In general, the proportions of sweet foods and beverages and fatty and fried foods were higher in middle-aged groups than in young groups. In contrast, refined carbohydrates intake was higher in young-aged groups than in middle-aged groups.

In young groups, obesity prevalence was significantly higher in women than in men. Similar significant outcomes were found in a middle-aged group. Figure 1 shows the significant differences in the prevalence of obesity (all *p* < 0.0001). Moreover, the prevalence of obesity increased by age. In the group aged 46–55 years, the prevalence of obesity slightly decreased in both men and women. In all age groups, the prevalence of obesity was significantly higher in women than in men. (Figure 2).

**Figure 3:**
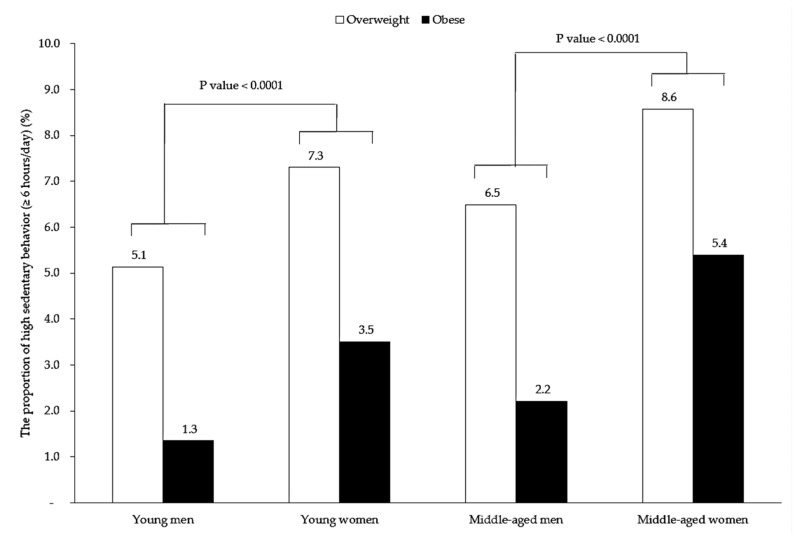
The proportion of high sedentary behaviors by gender and age.

Furthermore, there were significant differences (*p* < 0.0001) in the proportions of high sedentary behaviors (inactivity) between young overweight and obese men and women and among middle-aged overweight and obese men and women (Figure 3).

**Figure 4:**
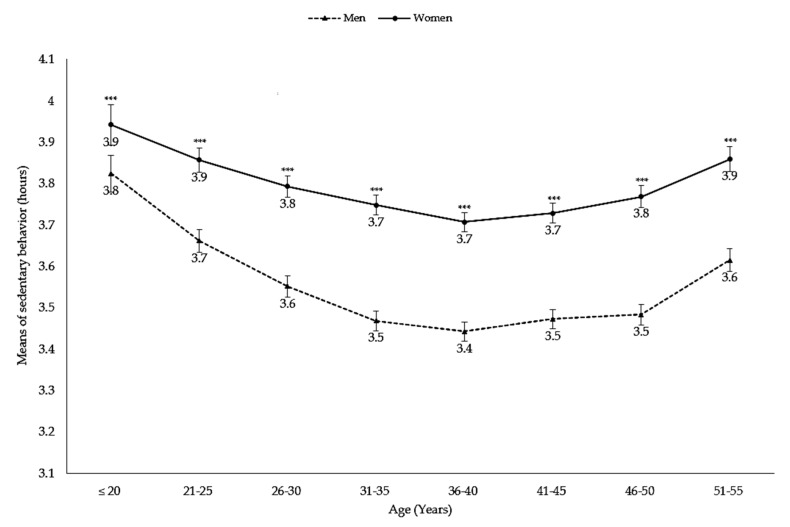
Mean hours of sedentary behaviors by age. Footnote: ***: *p* < 0.0001.

The mean duration of sedentary behaviors decreased in the group aged 20–40 years and increased after 40 years of age. In all age groups, significant differences were found between men and women (*p* < 0.0001) (Figure 4).

Moreover, there were significant differences (*p* < 0.0001) in the refined carbohydrates, sweet foods and beverages, and fatty and fried foods intakes between men and women in the young, overweight and obese groups and between men and women in the middle-aged, overweight and obese groups (Supplemental Figures S1–S3).

The association of sedentary behavior and unhealthy foods intake with obesity was estimated using Poisson regression (Table 2 and Table 3). Age, sedentary behaviors, and intakes of refined carbohydrates, fatty and fried foods, and sweet foods and beverages increased the obesity risk both in men and in women. To investigate the risk factors of obesity, we analyzed the main effect and related interaction of gender using Multivariate Analysis (Table 3). It was found that women had a two times higher adjusted prevalence ratio (PR) of obesity than men in five models. There were positive multiplicative interactions between gender and fatty and fried foods with obesity only in women, suggesting that, in women, fatty and fried foods increase the risk of obesity more than in men (Interaction prevalence ratio = 1.05, 95% CI = 1.02–1.09). In contrast, negative multiplicative interactions between gender and sedentary behaviors and refined carbohydrates intake were observed in women. In fact, in women, a less multiplicative (but still additive) effect on obesity risk was found for sedentary behavior (0.93, 0.90–0.97) and refined carbohydrates intake (0.93, 0.89–0.97). In addition, the effects of sweet foods and beverages and of their interaction with gender were not significant. In the final model, the main effects of gender, sedentary behaviors, unhealthy foods intake, and their interactions were stable and significant.

## 4. Discussion

In this population-based study, we identified strong relationships between obesity risk and sedentary behaviors and unhealthy foods, especially in women. This study also showed the impact of gender in relation to sedentary behaviors and unhealthy foods on obesity risk. After adjustment for age and education, the women gender’s effect on obesity persisted and was significant compared to men. There was also a positive and significant interaction between obesity and sedentary behaviors and unhealthy foods intake. However, fatty and fried foods displayed a positive multiplicative interaction in women more than in men, indicating a possible dietary risk for women in terms of obesity.

The prevalence of adulthood obesity increased of 4.1% (24.6% in 2007 to 28.7% in 2010) in women and of 2.2% (14.8% in 2007 to 17% in 2010) in men in three years [3]. In 2013, the prevalence of obesity was still higher in women (32.9%) than in men (19.7%) [22]. This means that there was a dramatic increase from 2010 to 2013, corresponding to 4.2% in women and 4.9% in men [22]. Data from the Indonesian Family and Life Surveys (IFLSs) also showed that the prevalence of women’s obesity rapidly increased from 1993 to 2007 by about 10% (9.67% to 19.64%) [4]. The highest proportion of adult obesity was in middle-aged women, especially those with aged 41–45 years (24.83%). This proportion was twice as that of men (11.95%) of the same age.

In our study, before adjusting for unhealthy foods intake, the obesity risk for women seemed to increase more than for men as a consequence of sedentary behaviors (Table 3). The mean duration of sedentary behaviors in obese men and women was 3.68–3.79 h/day and 3.90–3.97 h/day, respectively. Figure 3 shows that the mean duration for men was lower than for women. A previous study also found that women were more likely to have higher sedentary behaviors than men [25] and identified less educated women, housewives, and widowed/divorced women with especially high sedentary behaviors [25]. One similar population-based case control study involving women aged 20–74 years found that sedentary behaviors may be associated with reduced estrogen metabolism [26]. Another study showed no association between high physical activity and lower obesity risk in women [27].

Moreover, fatty and fried foods indicated a positive multiplicative effect in obesity risk in women in Table 3. Obese Indonesian women of reproductive age also had unhealthy dietary patterns characterized by consumption of oils and fats through fried foods and snacks [28,29]. This dietary pattern may contribute to a higher risk of obesity among women. Those who had a higher intake of refined carbohydrates also had a higher prevalence ratio of obesity; however, the interaction between gender and refined carbohydrates intake showed a negative multiplicative interaction. Some other related studies showed that sweet foods and beverages were significant risk factors for obesity [28,29,30,31,32,33]. It was also found in one study that one additional spoonful of sugar consumed every day increased the risk of being overweight and obese by about 14% [33]. In contrast, our study did not show a significant association between sweet foods and beverages and obesity.

Our study showed that women had a higher obesity risk than men even if they consumed refined carbohydrates or fatty fried foods with the same frequency. However, gender differences regarding the relationship between dietary habits and obesity are inconsistent. The biological actions of endogenous estrogen may also be influenced by macronutrient intake [34]. Estrogen was associated with changes in body weight in women [35]. Interactions between estrogen, leptin, and thyroid hormones regulate the energy expenditure [36]. Moreover, the energy expenditure in women was lower than in men, thus fat storage tended to be higher in women [37]. Therefore, women should be more susceptible to obesity than men. Furthermore, culture-related factors are also important and should also be considered. The basic daily dishes from breakfast to dinner among Indonesian people are not varied and consist of steamed rice, a hot fried dish, and a coconut milk dish [38].

The strength of this study is that we used a large and representative sample of the Indonesian population. The questionnaires about physical activity and dietary behaviors were specific, and additional physical activity and dietary intake cards were utilized. Therefore, the reporting bias from the participants was minimized. Second, this study is the first in Indonesia exploring the prevalence of obesity and the associations between obesity and sedentary behaviors and unhealthy foods intake in the adult population.

However, one of the limitations of this study is the cross-sectional design. Further research should consider a prospective cohort study to explore the different effects of sedentary behaviors and unhealthy foods on obesity, especially in women. Also, we were unable to explain the different mechanisms leading to obesity because of the unavailability of additional data. For example, we were unable to find the exact weight of foods consumed (gram), the energy intake (in kcal), and the metabolic equivalents of the different activities. Moreover, we could not include some confounders, such as co-morbidities or estrogen levels.

## 5. Conclusions

Based on the Indonesian Basic Health Research Survey data, this study reports an increase in obesity prevalence, high sedentary behaviors, and unhealthy foods intake especially in women. Our findings also suggest that unhealthy intake of fatty fried foods positively interacts with gender to increase the prevalence ratio of obesity in women more than in men. Future investigations should consider longitudinal studies to explore the different effects on obesity of sedentary behaviors and dietary patterns in the two genders. Nutritional and physical activity educational programs should be conducted, as they can play an important role in improving the physical activity levels, developing healthy eating behaviors, and maintaining a healthy lifestyle.

## Figures and Tables

**Table 1:** Characteristics of the study participants according to age and gender.

Variables	Young Age				*p* Value	Middle-Age				*p* Value
	Men	%	Women	%		Men	%	Women	%	
**Body Mass Index (BMI)**										
Mean (SD)	22.6 (3)		23.7 (3.7)			23.5(3.2)		25 (3.9)		
**Education**										
College Graduate	8581	8.9	11,703	10.6	<0.0001	11,938	9.5	10,471	7.6	<0.0001
Not College Graduate	88,049	91.1	98,535	89.4		114,082	90.5	127,881	92.4	
**Nutritional Status by BMI**										
Normal Weight	60,970	63.1	56,448	51.2	<0.0001	62,992	50.0	48,351	34.9	<0.0001
Overweight	29,195	30.2	37,292	33.8		48,532	38.5	56,776	41.0	
Obese	6465	6.7	16,498	15.0		14,496	11.5	33,225	24.0	
**Sedentary Behavior**										
<3 h/d	39,376	40.7	42,869	38.9	<0.0001	53,541	42.5	54,699	39.5	<0.0001
3–5 h/d	40,730	42.2	44,122	40.0		51,864	41.2	55,208	39.9	
≥6 h/d	16,524	17.1	23,247	21.1		20,615	16.4	28,445	20.6	
**Refined Carbohydrates**										
<1x/day	82,609	85.5	91,055	82.6	<0.0001	108,541	86.1	116,218	84.0	<0.0001
≥1x/day	14,021	14.5	19,183	17.4		17,479	13.9	22,134	16.0	
**Sweet Foods and Beverages**										
<1x/day	43,422	44.9	56,016	50.8	<0.0001	54,570	43.3	66,943	48.4	<0.0001
≥1x/day	53,208	55.1	54,222	49.2		71,450	56.7	71,409	51.6	
**Fatty and Fried Foods**										
<1x/day	66,329	68.6	72,336	65.6	<0.0001	85,479	67.8	87,641	63.3	<0.0001
≥1x/day	30,301	31.4	37,902	34.4		40,541	32.2	50,711	36.7	

*p* values were from the Chi-squared test. Normal weight: BMI: 18.5–23 kg/m^2^; overweight: 23.0–27.5 kg/m^2^; obese: ≥27.5 kg/m^2^.

**Table 2:** Prevalence ratio (PR) of obesity by gender.

Variable	PR	95% CI	*p* Value
Men			
Age (Middle-aged vs. young)	1.71	(1.66–1.76)	<0.0001
Education (Graduate vs. non-graduate)	0.49	(0.47–0.51)	<0.0001
Sedentary Behavior (≥6 h vs. <6 h/d)	1.18	(1.14–1.22)	<0.0001
Refined Carbohydrates (≥1x vs. <1x/day)	1.18	(1.14–1.23)	<0.0001
Sweet foods and Beverages (≥1x vs. <1x/day)	0.98	(0.96–1.01)	0.2278
Fatty and Fried Foods (≥1x vs. <1x/day)	1.08	(1.05–1.12)	<0.0001
Women			
Age (Middle-aged vs. young)	1.61	(1.58–1.64)	<0.0001
Education (Graduate vs. non-graduate)	0.92	(0.90–0.95)	<0.0001
Sedentary Behavior (≥6 h vs. <6 h/d)	1.11	(1.09–1.13)	<0.0001
Refined Carbohydrates (≥1x vs. <1x/day)	1.15	(1.12–1.17)	<0.0001
Sweet foods and Beverages (≥1x vs. <1x/day)	0.97	(0.95–0.99)	0.0005
Fatty and Fried Foods (≥1x vs. <1x/day)	1.14	(1.12–1.16)	<0.0001

Data are presented as prevalence ratio (PR) with 95% CI.

**Table 3:** PR of obesity with respect to sedentary behaviors, intakes of refined carbohydrates, sweet foods and beverages, and fatty and fried foods, and their interactions.

Variable (Risk vs. Reference)	PR	95% CI	*p* Value
Model 1			
Gender (Women vs. Men)	2.16	2.12–2.20	<0.0001
Age (Middle-aged vs. young)	1.65	1.62–1.68	<0.0001
Education level (Graduate vs. non-graduate)	0.72	0.71–0.74	<0.0001
Sedentary behavior (SB) (≥6 h vs. <6 h/d)	1.20	1.16–1.25	<0.0001
Gender × SB	0.93	0.90–0.97	0.0006
Model 2			
Gender (Women vs. Men)	2.16	2.12–2.20	<0.0001
Age (Middle-aged vs. young)	1.65	1.63–1.68	<0.0001
Education level (Graduate vs. non-graduate)	0.73	0.72–0.75	<0.0001
Refined carbohydrate intake (RCI) (≥1x vs. <1x/day) (Figure S1)	1.24	1.19–1.28	<0.0001
Gender × RCI	0.93	0.89–0.97	0.0004
Model 3			
Gender (Women vs. Men)	2.16	2.10–2.21	<0.0001
Age (Middle-aged vs. young)	1.65	1.62–1.68	<0.0001
Education level (Graduate vs. non-graduate)	0.72	0.70–0.74	<0.0001
Sweet Foods and Beverages (SFB) (≥1x vs. <1x/day)	1.01	0.99–1.04	0.3275
Gender × SFB	0.99	0.96–1.02	0.4328
Model 4			
Gender (Women vs. Men)	2.09	2.05–2.13	<0.0001
Age (Middle-aged vs. young)	1.65	1.62–1.67	<0.0001
Education level (Graduate vs. non-graduate)	0.72	0.70–0.74	<0.0001
Fatty& fried foods intake (FFFI) (≥1x vs. <1x/day)	1.10	1.07–1.13	<0.0001
Gender × FFFI	1.05	1.02–1.09	0.0045
Model 5			
Gender (Women vs. Men)	2.13	2.08–2.18	<0.0001
Age (Middle-aged vs. young)	1.65	1.62–1.68	<0.0001
Education level (Graduate vs. non-graduate)	0.73	0.72–0.75	<0.0001
SB (≥6 h vs. <6 h/d)	1.20	1.16–1.24	<0.0001
RCI (≥1x vs. <1x/day)	1.22	1.18–1.27	<0.0001
FFFI (≥1x vs. <1x/day)	1.07	1.04–1.10	<0.0001
Gender × SB	0.93	0.89–0.96	0.0002
Gender × RCI	0.92	0.88–0.96	0.0001
Gender × FFFI	1.06	1.02–1.10	0.0008

Data are presented as prevalence ratio (PR) with 95% CI.

## Supplementary Materials

The following are available online at http://www.mdpi.com/2072-6643/10/6/704/s1, Figure S1: Refined Carbohydrate Intake; Figure S2: Sweet Foods and Beverages Intake; Figure S3: Fatty and Fried Foods Intake.
