# Supplementary figures and images for: Roles of Sedentary Behaviors and Unhealthy Foods in Increasing the Obesity Risk in Adult Men and Women: A Cross-Sectional National Study
Source: Nutrients. 2018 May 31;10(6):704. doi: 10.3390/nu10060704 (PMC6024814; doi:10.3390/nu10060704)

## Supplementary 1. Unhealthy Food Consumption among Obese Indonesian Men and Women.

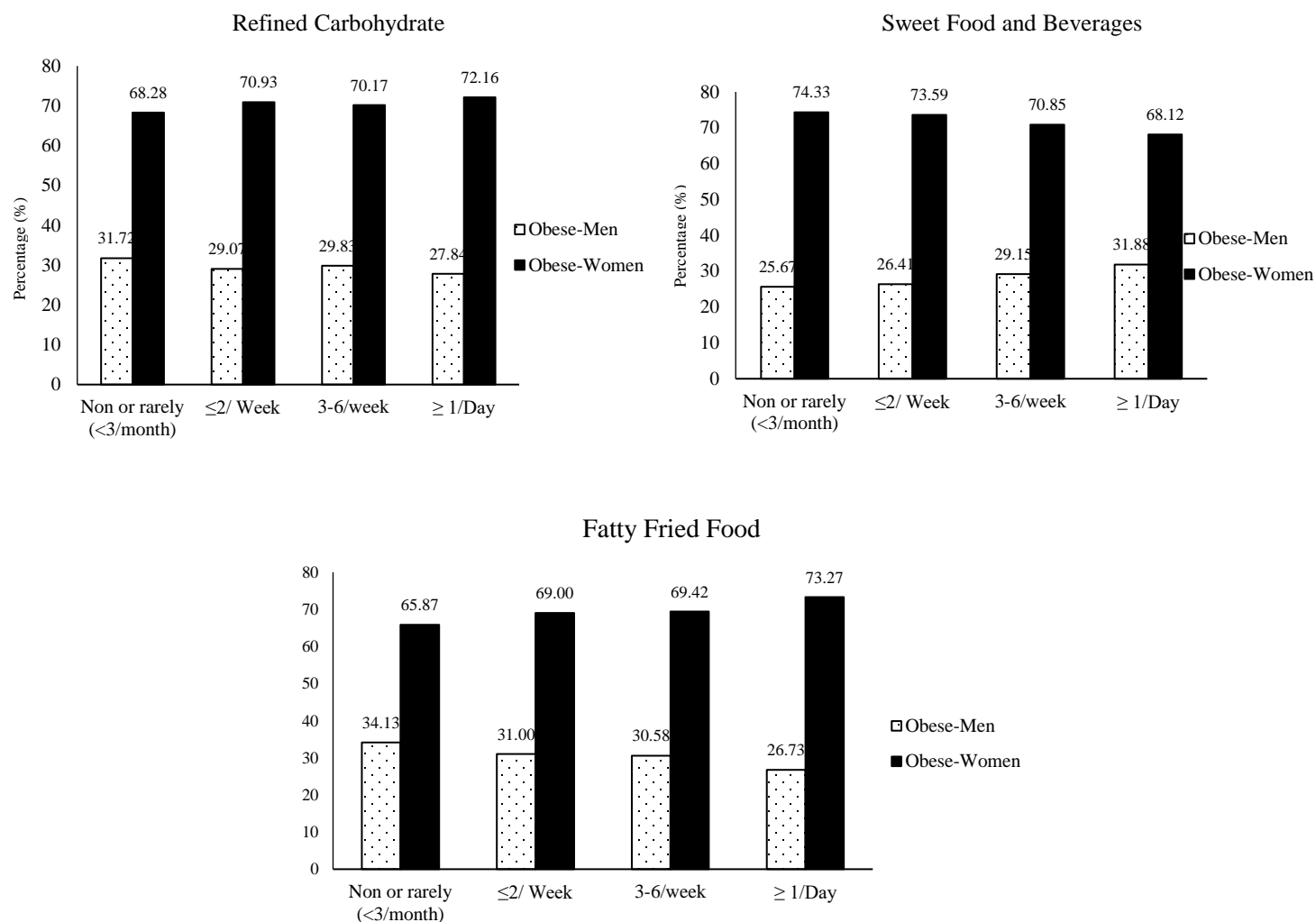

Supplement: Supplementary file 1 [file nutrients-10-00704-s001.pdf]
